# Impact of draining cannula position and configuration under high-flow extracorporeal CO_2_ removal (ECCO_2_R) on recirculation and extracorporeal decarboxylation

**DOI:** 10.1007/s10047-026-01582-7

**Published:** 2026-10-10

**Authors:** Jonathan Charbit, Clara Muller, Mehdi Girard, Elie Courvalin, Geoffrey Dagod, Karim Bouchdoug, Pauline Deras, Hugues Weber, Xavier Capdevila

**Affiliations:** Département d’Anesthésie Réanimation Lapeyronie, Critical Care Unit, Hôpital Lapeyronie, Lapeyronie University Hospital, 371 Avenue du Doyen G. Giraud, 34295 Montpellier Cedex 5, France

**Keywords:** CO_2_ clearance, CO_2_ elimination, Extracorporeal pump flow, Extracorporeal respiratory support, Decarboxylation index, High-flow ECCO_2_R, Inlet cannula, Inlet CO_2_ partial pressure, Inlet oxygen saturation

## Abstract

The main goal of the present study was to demonstrate the influence of draining cannulas’ position and high-flow extracorporeal CO_2_ removal **(**ECCO_2_R) configuration on recirculation and extracorporeal decarboxylation. This single-center retrospective study was conducted between January 2018 and May 2024. All patients who received a high-flow ECCO_2_R (extracorporeal blood flow ≥ 2 L/min) were included. Four configurations each used an ECMO device, two single-lumen cannulas, and an ipsilateral cannulation site: *CENTRAL DRAINAGE* configurations (*central jugular-jugular* and *central femoro-femoral*), *DISTAL DRAINAGE* configurations (*distal jugular-jugular* and *distal femoro-femoral*). Recirculation coefficient and CO_2_ elimination were assessed between these configurations. The influence of the configuration and recirculation coefficient on CO_2_ elimination was studied by multivariable mixed linear model. Eighteen patients were treated with high-flow ECCO_2_R. Extracorporeal blood flows (median of 2.6 L/min [IQR, 2.0‒2.6 L/min]) were comparable between the configurations (*P* = 0.33), although values higher than 2.5 L/min were observed in *CENTRAL DRAINAGE* configurations. Median CO_2_ elimination was 110 mL(STPD)/min (IQR, 83‒149 mL(STPD)/min). Recirculation coefficient was significantly higher with the *DISTAL DRAINAGE* configurations (59% [IQR, 51‒71%] *versus* 9% [IQR, 15‒22%], respectively; *P* < 0.001). Consequently, CO_2_ elimination was significantly lower with the *DISTAL DRAINAGE* configurations (58 mL(STPD)/min [IQR, 44‒72 mL(STPD)/min] *versus* 122 mL(STPD)/min [IQR, 96‒159 mL(STPD)/min], respectively; *P* = 0.003). Both *DISTAL DRAINAGE* configurations and high recirculation coefficient were significantly associated in multivariable analysis with a reduction in CO_2_ elimination (*P* < 0.001). We evaluated four different configurations of high-flow ECCO_2_R using single-lumen cannulas allowing either a central or a distal drainage. A central drainage configuration gave better rheological conditions and lower recirculation, leading to better extracorporeal decarboxylation.

**Graphical abstract:**

**Figure:**
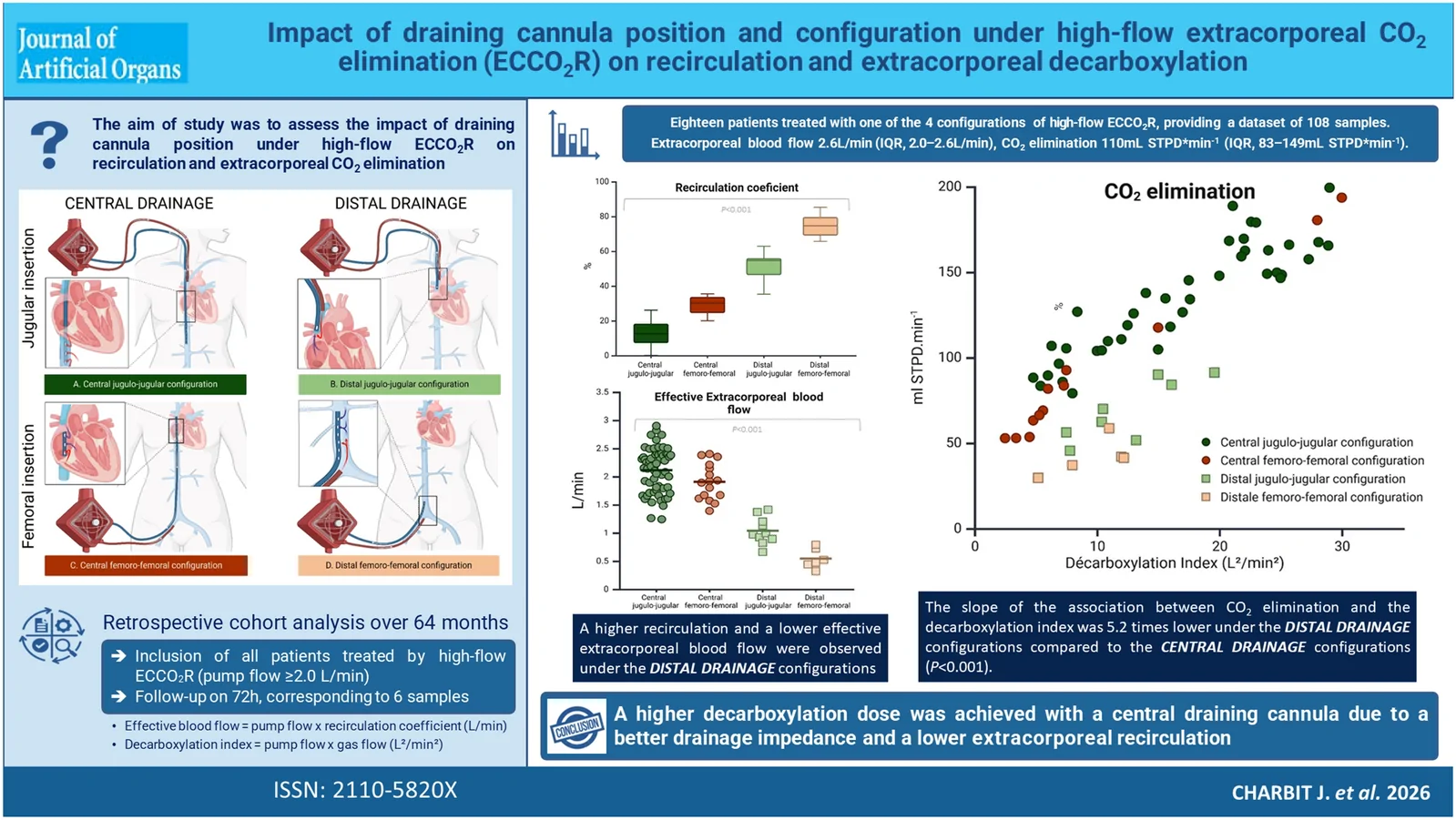

**Supplementary Information:**

The online version contains supplementary material available at https://doi.org/10.1007/s10047-026-01582-7.

## Background

Although first described in the late 1970s, extracorporeal CO_2_ removal (ECCO_2_R) remains a rarely used treatment in an intensive care setting [1, 2]. Prolonged hypercapnia has well-demonstrated deleterious effects [3, 4]. ECCO_2_R could play an important role in the rescue management of acute respiratory failure (ARF) [5, 6]. In patients suffering from acute respiratory distress syndrome (ARDS), ECCO_2_R would allow less aggressive mechanical ventilation and the use of ultraprotective pulmonary ventilation, thus reducing ventilator-induced lung injury [5]. Furthermore, ECCO_2_R would also allow a reduction in or avoidance of the need for intubation in cases of refractory respiratory failure such as exacerbations of chronic obstructive pulmonary disease (COPD) or respiratory failure associated with hypoventilation [5–7].

Initially based on pumpless arterio-venous extracorporeal lung assistance, ECCO_2_R has shifted to veno-venous centrifugal or diagonal rotary pumps, generating different pressure and flow regimens. Variations in devices, as well as in catheter diameters and configurations, have led to significant disparities in rheological conditions and decarboxylation performance [8]. The main determinants of extracorporeal decarboxylation have been demonstrated to include sweep gas flow, oxygenator surface, intrinsic properties, and inlet carbon dioxide (CO_2_) content [8–11]. The recirculation phenomenon could also have a significant impact on decarboxylation [12], but the wide diversity of devices and configurations has impeded its demonstration. Recently, extracorporeal blood flow has been highlighted as another major determinant of extracorporeal decarboxylation, distinguishing low-flow and high-flow ECCO_2_R systems [8, 12, 13]. The threshold defining “high-flow” is often established between 1000 and 1500 mL/min but is still debated [6, 14–17]. Recent trials support the concept that only veno-venous ECCO_2_R devices using high flow can successfully manage severe respiratory acidosis or significantly reduce mechanical ventilation parameters [10, 14, 15, 18, 19].

The kind of cannulas, their diameters, and the positioning of the draining cannula have a major influence on extracorporeal pump flow and the decarboxylation dose under ECCO_2_R therapy. However, these different parameters have not been studied directly to determine their impact on extracorporeal decarboxylation and its clinical consequences. Moreover, the use of single-lumen cannulas and central positioning has been poorly studied in this context. Therefore, it seemed relevant to assess rheological parameters and decarboxylation among different ECCO_2_R configurations using high extracorporeal blood flows and two single-lumen cannulas. In addition, we wished to study the specific consequences of draining cannula position on recirculation and extracorporeal decarboxylation.

The aim of this retrospective observational study was to assess the influence of draining cannula position and high-flow ECCO_2_R configuration on rheological conditions (pressures, flows, and recirculation) and their consequences for extracorporeal decarboxylation.

## Materials and methods

### Design and setting

After obtaining approval from the local ethics committee (*IRB number 202000662, Montpellier University Hospital*), this retrospective study was conducted in the critical care unit of Lapeyronie University Hospital in Montpellier between January 2018 and May 2024.

### Inclusion criteria

All patients in our unit who needed extracorporeal respiratory support and were treated during the study period with high-flow ECCO_2_R (extracorporeal blood flow ≥ 2.0 L/min) were retrospectively included. Exclusion criteria were the following: under 18 years of age, pregnancy, or ongoing juridical protection. Several of these patients had been included in previous studies focused on extracorporeal decarboxylation dose and biochemical mechanisms across the extracorporeal oxygenator [19].

### Management of patients and ECCO_2_R configurations

**Fig. 1:**
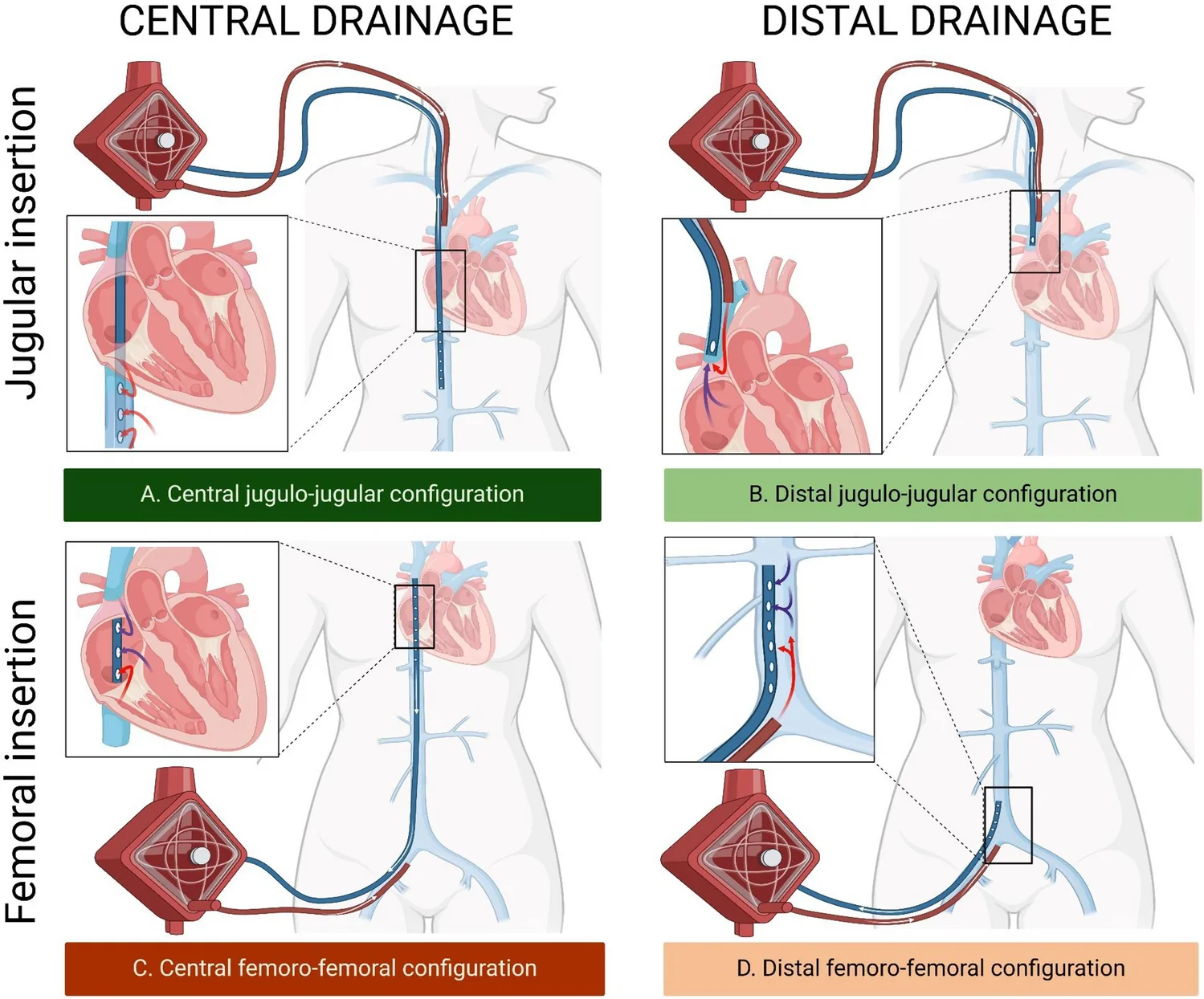
Different high-flow ECCO_2_R configurations. By using two separate single-lumen cannulas, the insertion site may be distinguished from the drainage area. We chose to prioritize a single insertion site by performing ipsilateral cannulation, in order to simplify the configuration and reduce the number of venous accesses required. In the CENTRAL DRAINAGE configurations, the draining cannula is a multistage cannula that will be placed through the right atrium to achieve an optimal drainage and minimize the recirculation phenomenon. A control via transthoracic ultrasonography (subxiphoid window) allows visualization of the guidewire, ensuring safe progression and optimal placement of the draining cannula across the right atrium. In central jugular-jugular configuration (**A**), the draining cannula, introduced into the right internal jugular vein, will terminate in the inferior vena cava and preferentially drain the inferior caval venous return. In central femoro-femoral configuration (**B**), the draining cannula, introduced in the femoral vein, will terminate in the superior vena cava, passing through the right atrium. A multistage draining cannula will drain the right atrium and even the inferior vena cava, generating an acceptable recirculation phenomenon. However, the quality of draining conditions is optimal and justifies this configuration. In the DISTAL DRAINAGE configurations, the draining cannula will be positioned close to the reinfusion cannula. To achieve a stable and high extracorporeal flow, it is preferable to place the draining cannula more proximally, regardless of the recirculation phenomenon

Patients who presented with refractory hypoxemia in our unit were treated by an ECMO configuration according to usual criteria. In contrast, a high-flow ECCO_2_R strategy was chosen for patients who needed extracorporeal respiratory support with acceptable hypoxemia. High-flow ECCO_2_R was implemented using an ECMO device (CardioHelp HLS 5.0 oxygenator [Maquet®; surface 1.3 m^2^] or X-Lung™ 230 oxygenator [Novalung®; surface 1.9 m^2^]) and two single-lumen cannulas (3/8″ connection) placed at the same puncture site (ipsilateral insertion). These devices were unchanged during the study period. Because of their presumed modest performance, dual-lumen catheters were not used in our unit in accordance with the team protocol. Schematically, four ECCO_2_R configurations were defined based on insertion site and draining cannula position: two with central drainage (the *central jugular-jugular configuration* and the *central femoro-femoral configuration*), and two with distal drainage (the *distal jugular-jugular configuration* and the *distal femoro-femoral configuration)* (Fig. 1). The choice of configuration was left to the clinician’s discretion according to different criteria. Femoro-femoral configurations were the first-line choice in awake patients with spontaneous breathing, while jugular-jugular configurations were the first-line choice in ventilated patients for whom decarboxylation objectives were high. Local venous limitations or thromboses could also influence the decision-making process for ECCO_2_R configuration.

All high-flow ECCO_2_R procedures were performed under sterile conditions in the ICU at the patient’s bedside. Cannulas were placed percutaneously according to the *Seldinger* technique with increasing dilator size. Ultrasonography was used for pre-mapping and to localize the guidewire and cannula insertion. Transthoracic echocardiography (subxiphoid view) was systematically used to visualize the right atrium or *inferior vena cava*. All draining cannulas had a multi-stage access on the distal 10-cm segment, except for the *distal jugular-jugular configuration.* The minimal extracorporeal pump flow was set at 2.0 L/min to limit the risk of premature alteration of the membrane. It could be increased if the decarboxylation dose was insufficient. The coagulation status was maintained within the target range in absence of contraindications (target APTT ratio between 1.5 and 2.0). Thereafter, when the respiratory conditions improved significantly, the decarboxylation dose could be decreased and ECCO_2_R weaning could start. When judged possible, extubation was attempted prior to ECCO_2_R explantation in order to limit the adverse consequences of sedation and mechanical ventilation. Otherwise, a thoraco-abdomino-pelvic CT scan was systematically performed after ECCO_2_R cannula elimination in order to diagnose thrombo-embolic events.

### Data collection

In our study, data were abstracted retrospectively from medical records and intensive care unit (ICU) records: demographics, admission Simplified Acute Physiology Score II (SAPS II), occurrence of organ failure (renal replacement therapy or ARDS) during the stay, indications for and characteristics of extracorporeal respiratory support and related complications. Mechanical ventilatory parameters, ECCO_2_R therapy parameters, and biological data were extracted every 12 h from ICU monitoring. Inlet and outlet blood gases were sampled every 12 h according to the unit protocol (through side ports on both sides of the oxygenator) and analyzed (ABL90 Flex Plus Radiometer, Copenhagen, Denmark). To minimize the consequences of extracorporeal membrane alteration on decarboxylation, the data analysis of extracorporeal CO_2_ elimination focused on the first six values, corresponding to the first threedays. The durations of mechanical ventilation and extracorporeal respiratory support therapy, length of ICU and hospital stays, and survival at discharge were also established for each patient.

### Study definitions

*CENTRAL DRAINAGE* configurations of high-flow ECCO_2_R included the *central jugular-jugular* and *central femoro-femoral* configurations, while *DISTAL DRAINAGE* configurations included the *distal jugular-jugular* and *distal femoro-femoral* configurations.

Different extracorporeal parameters were calculated as follows:

- The decarboxylation index in L^2^/min = extracorporeal blood flow × fresh gas flow [16].
- Recirculation (R) coefficient as a percentage = (S_in_O_2_–SvO_2_)/(S_out_O_2_–SvO_2_), where S_in_O_2_ is the oxygen saturation in the inlet blood circulation, S_out_O_2_ is the oxygen saturation in the blood from the outlet blood circulation, and S_V_O_2_ is the oxygen saturation of blood in the pulmonary artery. Because SvO_2_ is not reliably available under extracorporeal respiratory support, an a priori value of 65% was used as the default.
- Effective extracorporeal blood flow in L_/_min, obtained by multiplying extracorporeal blood flow × (1 − R coefficient).
- CO_2_ elimination through the membrane oxygenator in mL(STPD)/min, obtained using the standard formula considering the difference in CO_2_ content between the inlet and the outlet extracorporeal circulation multiplied by blood flow (*Supplemental Digital Content 1*).

Membrane dysfunction, indicated by a significant coagulopathy and fibrinogen consumption, resolved with a change of extracorporeal lung membrane [20]. Cannula-associated deep vein thrombosis was defined as a deep vein thrombosis diagnosed at cannulation site.

### Statistical analysis

The main characteristics of the included patients were detailed and compared according to the ECCO_2_R configuration. The two *DISTAL DRAINAGE* configurations were grouped because of their low number. Categorical variables were reported as numbers (percentage) and ontinuous variables were reported as medians (interquartile ranges [IQR]). The study analysis next focused on the rheological conditions (inlet/outlet pressures, S_in_O_2_, extracorporeal blood flow, and recirculation) of the different high-flow ECCO_2_R configurations. A stratification of values used a priori thresholds of the R coefficient. Considering the paired nature of the data, linear mixed models were used for the statistical analysis of these variables according to the ECCO_2_R configuration, as well as according to the R coefficient level. In each model, either the ECCO_2_R configuration (*central jugular-jugular* or *CENTRAL DRAINAGE* as reference) or the R coefficient level (< 20% as reference) was considered a fixed effect, while the intercept and the patient were included as random effects. To strengthen the reliability of our analysis and account for varying clinical SvO_2_ values, a sensitivity analysis was conducted using different assumed SvO_2_ values within a range of 60–70% for the calculation of the recirculation coefficient.

Finally, three other linear mixed models were built to highlight the impact of the ECCO_2_R configuration and recirculation on extracorporeal decarboxylation. The linear relationship between the decarboxylation index and CO_2_ elimination demonstrated in a previous study [19] was thus assessed by these multivariable models, considering CO_2_ elimination as the dependent variable. The explanatory variables selected a priori for the present analysis were the patient, admission SAPS II score, ECCO_2_R configuration, oxygenator type and membrane surface area, hospital day at ECCO_2_R implementation, the R coefficient and the decarboxylation index. Because their influence may be considered proportional, the interaction terms decarboxylation index _*_ ECCO_2_R configuration (*central jugular-jugular* or *CENTRAL DRAINAGE* as reference) and decarboxylation index _*_ R coefficient level (< 20% as reference) were alternatively included as fixed effects. The slope and the patient were included as random effects. Statistical significance was determined by *P* < 0.05. Statistical analysis was conducted using SAS® online software.

## Results

### Patient characteristics and ECCO_2_R configurations

During the study period, 18 patients were treated in our unit with high-flow ECCO_2_R; one patient met the exclusion criteria (missing data). No patients were excluded because of death occurring within 24 h after ECCO_2_R initiation. Among 17 studied patients, the median age was 51 years (IQR, 43‒59 years); 13 (76%) were male. Causes for hospitalization were COPD exacerbation (5/17, 29%), severe trauma (5/17, 29%), viral pulmonary infection (3/17, 18%), bacterial pneumonia (2/17, 12%), or other etiologies (2/17, 12%). The median SAPS II score on admission was 41 (IQR, 27‒71). The main characteristics of the cohort are summarized in Table 1.

**Table 1:** Admission characteristics and outcome according to configuration

	*Central Jugulo-Jugular* configuration	*Central Femoro-Femoral* configuration	*DISTAL DRAINAGE* configuration	Total
	*n* = 11	*n* = 3	*n* = 3	*N* = 17
Age (years)	47 (42‒52)	62 (56‒68)	53 (47‒59)	51 (43‒59)
*Sex, n* (%)				
Male	9 (82%)	3 (100%)	1 (33%)	13 (76%)
Female	2 (18%)	0	2 (67%)	4 (24%)
*Reasons for Hospitalization*, *n* (%)				
COPD exacerbation	2 (18%)	2 (66%)	1 (33%)	5 (29%)
Severe trauma	3 (27%)	0	2 (66%)	5 (29%)
Viral pulmonary infection	2 (18%)	1 (33%)	0	3 (18%)
Bacterial pneumonia	2 (18%)	0	0	2 (12%)
Other	2 (18%)	0	0	2 (12%)
**SAPSII**	49 (27‒71)	45 (39‒49)	54 (40‒70)	41 (27‒71)
*ECCO*_*2*_*R and ventilation management*				
ECCO_2_R duration (days)	13 (5‒14)	8 (5‒11)	5 (3‒6)	8 (3.5‒13)
Mechanical ventilation duration (days)	26 (15‒42)	7 (2‒10)	15 (5‒22)	16 (5‒37)
Mechanical ventilation on day 1, *n* (%)	11 (100%)	1 (33%)	3 (100%)	15 (88%)
Extubation under ECCO_2_R, *n* (%)	4 (36%)	1 (33%)	1 (33%)	6 (35%)
Extubation failure under ECCO_2_R, *n* (%)	0	1 (33%)	0	1 (6%)
Extubated after decannulation, *n* (%)	2 (18%)	1 (33%)	1 (33%)	4 (24%)
Tracheotomy, *n* (%)	3 (27%)	0	1 (33%)	4 (24%)
Conversion to an ECMO, *n* (%)	2 (18%)	0	1 (33%)	2 (12%)
*Organ failure*				
Renal replacement therapy, *n* (%)	3 (27%)	1 (33%)	1 (33%)	5 (29%)
PaO_2_/F_i_O_2_ ratio at day 1	164 (123‒197)	181 (124‒220)	159 (156‒163)	156 (124‒195)
*Complications*				
Thrombo-embolic events, *n* (%)	7 (64%)	2 (67%)	0	8 (47%)
Femoral vein	1 (9%)	0	0	1 (6%)
Iliac vein	2 (18%)	1 (33%)	0	3 (18%)
Inferior *vena cava*	1 (9%)	2 (66,7%)	0	3 (18%)
Superior *vena cava*	1 (9%)	0	0	1 (6%)
Intern jugular vein	7 (64%)	0	0	7 (41%)
Bleeding related to cannulas, *n* (%)	1 (9%)	0	1 (33%)	2 (12%)
Membrane lung dysfunction, *n* (%)	3 (27%)	0	0	3 (18%)
Local infections, *n* (%)	0	0	0	0
*Outcome*				
ICU hospitalization (days)	38 (22‒50)	19 (12‒23)	20 (6‒27)	32 (12–49)
Total hospitalization (days)	45 (33‒55)	24 (21‒27)	27 (17‒36)	46 (18–51)
Mortality, *n* (%)	4 (36%)	0	1 (33%)	5 (29%)

Data are presented as data (percentage) or median (IQR) as appropriate

COPD, chronic obstructive pulmonary disease; ECCO2R, extracorporeal carbon dioxide removal; ECMO, extracorporeal membrane oxygenation; ICU, intensive care unit; PaO2/FiO2; ratio partial pressure of oxygen in arterial blood divided by the fraction of inspired oxygen; SAPSII simplified acute physiology score II

The median day of high-flow ECCO_2_R implantation was 3 days (IQR, 0‒7 days). Fifteen patients (88%) were under mechanical ventilation. Initial high-flow ECCO_2_R configurations were the following: 11 (65%) were *central jugular-jugular*, two (12%) were *distal jugular-jugular,* three (18%) were *central femoro-femoral,* and one (5%) was *distal femoro-femoral*. Therefore, 14 (82%) were *CENTRAL DRAINAGE* configurations and three (18%) were *DISTAL DRAINAGE* configurations. Details of the used cannulas and devices are summarized in in Table 2 and the *Supplemental Digital Content* 2.

### Rheological parameters and ECCO_2_R configurations

**Fig. 2:**
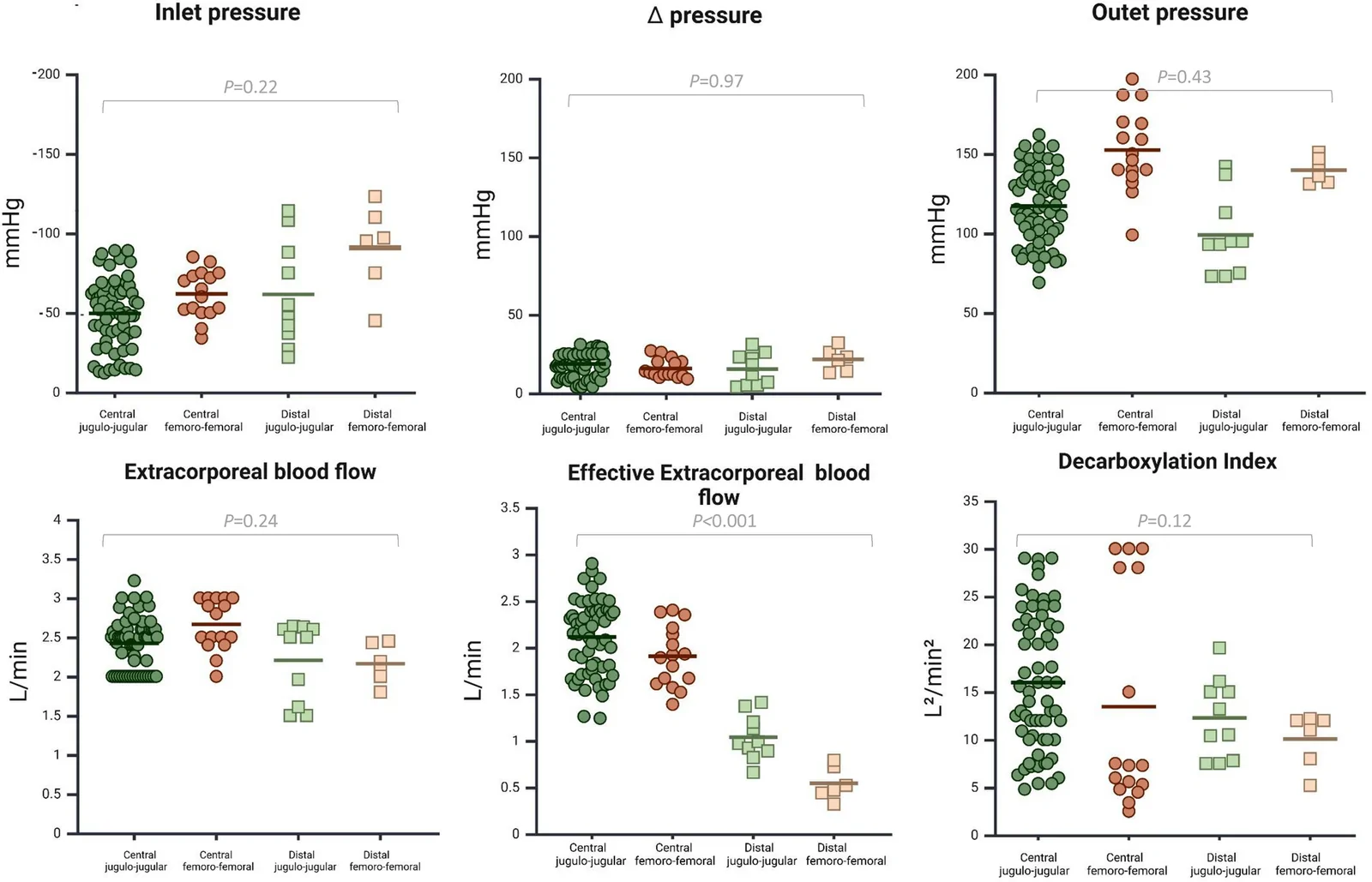
Rheological conditions according to ECCO_2_R configuration. Despite higher extracorporeal blood flows in the CENTRAL DRAINAGE configurations, close to 3 l/min, inlet pressures were slightly lower. Decarboxylation index, product of the extracorporeal blood flow by the fresh gas flow, were therefore higher in the CENTRAL DRAINAGE configurations. Finally, a higher recirculation phenomenon in DISTAL DRAINAGE configurations led to higher effective extracorporeal blood flows CENTRAL DRAINAGE configurations (*P* > 0.001)

Median extracorporeal blood flow of the cohort in the dataset was 2.6 L/min (IQR, 2.0‒2.6 L/min). This value did not significantly differ according to the type of high-flow ECCO_2_R configuration (*P* = 0.34). However, higher extracorporeal blood flows were observed in *CENTRAL DRAINAGE* configurations, in which all values were higher than 2.5 L/min (Fig. 2). Regarding pressures of extracorporeal circulation, inlet pressures tended to be lower in *CENTRAL DRAINAGE* configurations in comparison to *DISTAL DRAINAGE* configurations (*P* = 0.09). Outlet pressures were significantly higher in femoro-femoral configurations (*P* < 0.003).

**Fig. 3:**
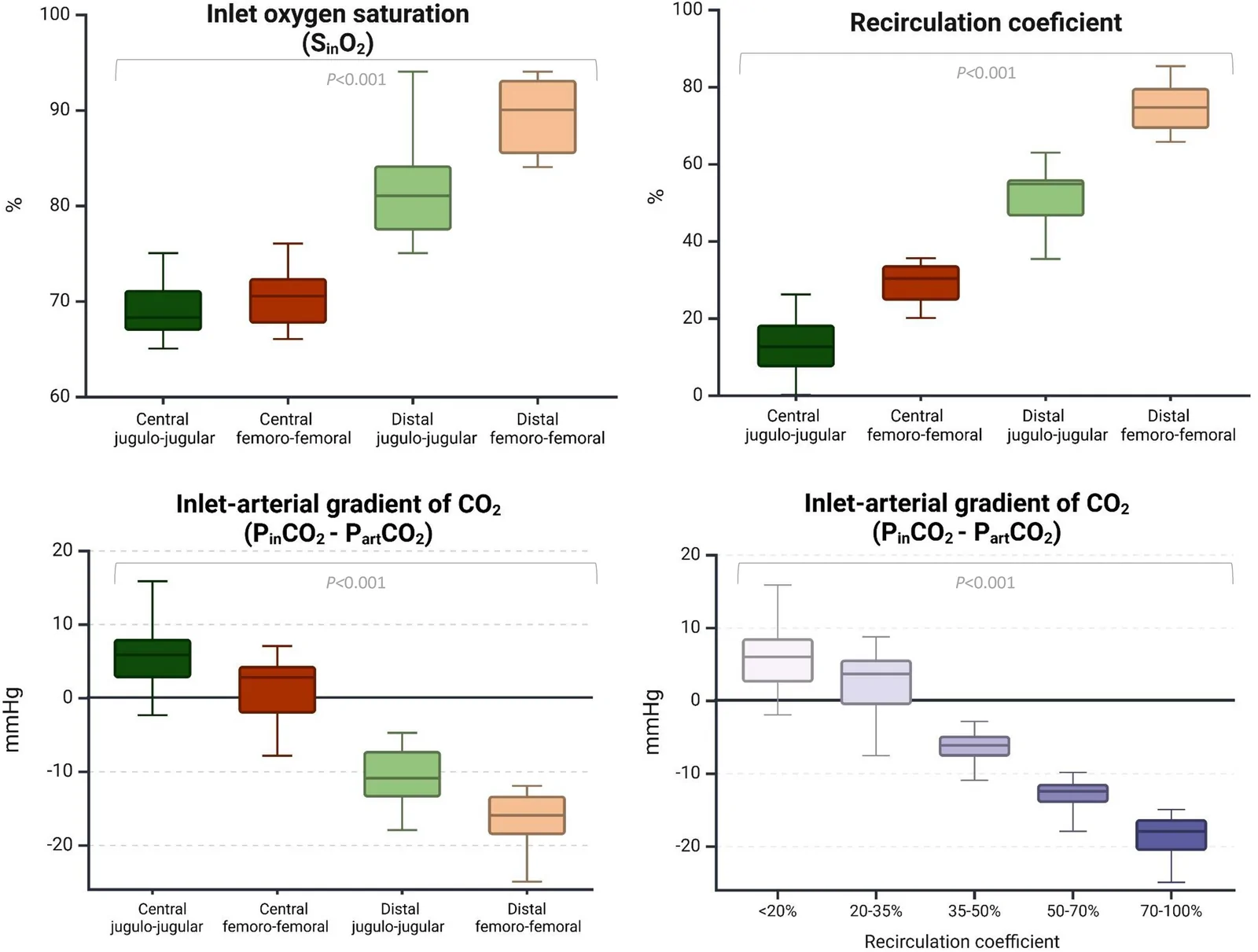
Recirculation phenomenon according to ECCO_2_R configuration. The DISTAL DRAINAGE configurations were observed with a higher recirculation than the CENTRAL DRAINAGE configurations. Direct consequences are a higher value of inlet oxygen saturation and a negative value in inlet-arterial gradient of P_CO2_. Regarding recirculation phenomenon, the central jugular-jugular configuration leads to inferior vena cava-to-right atrium route, which is known to be optimal. The central femoro-femoral configuration allows an acceptable capitation of blood reinfused by ECCO_2_R system, making this configuration a relevant compromise. The two DISTAL DRAINAGE configurations, in contrast, are associated with high values of recirculation explainable by a modest impedance of drainage into the distal areas

Median S_in_O_2_ in the cohort was 69% (IQR, 67‒72%), revealing a median R coefficient of 18% (IQR, 9‒30%). As expected, S_in_O_2_ and R coefficient were significantly higher in the *DISTAL DRAINAGE* configurations than in the *CENTRAL DRAINAGE c*onfigurations: 79% (IQR, 84‒90%) *versus* 68% (IQR, 67‒71%; *P* < 0.001), and 59% (IQR, 51‒71%) *versus* 9% (IQR, 15‒22%; *P* < 0.001), respectively (Fig. 3). Higher R coefficients corresponded to higher inlet-arterial gradients of partial pressure of CO_2_ (*P* < 0.001) (Fig. 3 and *Supplemental Digital Content 3*). Considering the recirculation phenomenon, effective extracorporeal blood flow was significantly lower in the *DISTAL DRAINAGE* configurations than in the *CENTRAL DRAINAGE c*onfigurations (0.9 L/min [IQR, 0.6‒1.1 L/min] *versus* 2.1 L/min [IQR, 1.7‒2.4 L/min], *P* < 0.001; Fig. 2), especially with the *distal femoro-femoral* configuration, for which the median value was 0.5 L/min (IQR, 0.4‒0.8 L/min). Sensitivity analysis using different assumed SvO_2_ values highlighted consistent trends in the relationship between the R coefficient and configurations (*Supplementary Digital Content* 4).

### Recirculation and CO_2_ elimination

**Fig. 4:**
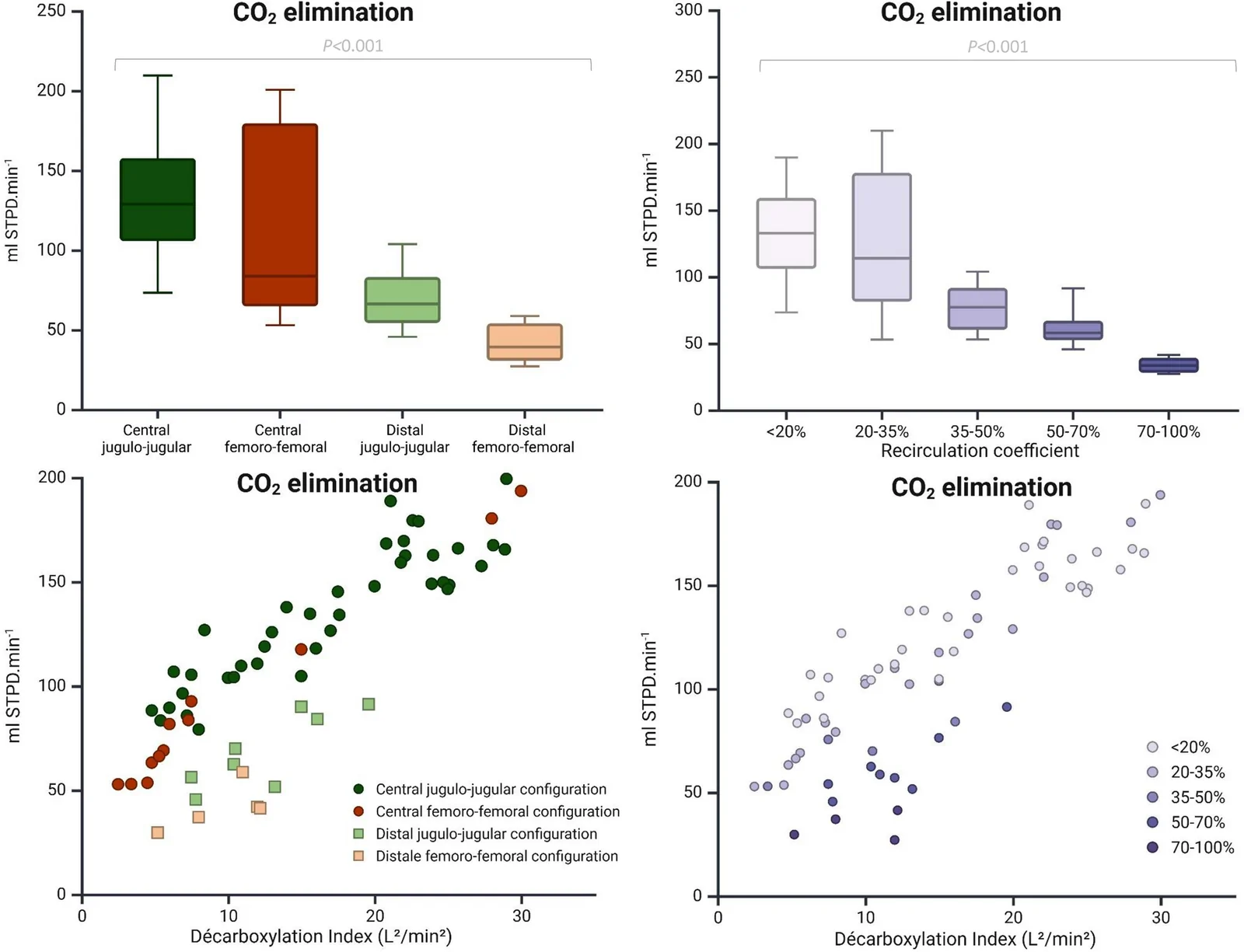
Impact of the configuration and recirculation on CO_2_ elimination. CO_2_ elimination is directly influenced by high-flow ECCO_2_ configuration and degree of recirculation. A distal drainage, which generates higher recirculation, was associated with a lower CO_2_ elimination for the same decarboxylation dose (couple gases/blood flows); the slope of the correlation line was correspondingly lower as the recirculation increased (See Supplemental Digital Content 3)

Median CO_2_ elimination by high-flow ECCO_2_R in our cohort was 110 mL(STPD)_/_min (IQR, 83‒149 mL(STPD)/min). *DISTAL DRAINAGE* configurations were significantly associated with lower CO_2_ elimination than *CENTRAL DRAINAGE* configurations (58 mL(STPD)/min [IQR, 44‒72 mL(STPD)/min] *versus* 122 mL(STPD)/min [IQR, 96‒159 mL(STPD)/min], respectively, *P* = 0.003). Based on the same phenomenon, high values of R coefficient were associated with a lower CO_2_ elimination (*P* < 0.001; Fig. 4). With an R coefficient of 35% or more, no observed value of CO_2_ elimination was higher than 100 mL(STPD)/min. Sensitivity analysis highlighted the same statistical association between the R coefficient and CO_2_ elimination, regardless of the assumed SvO_2_ value (*Supplementary Digital Content* 4).

The multivariable linear mixed regression analysis demonstrated that for a given decarboxylation index, both ECCO_2_R configuration and the R coefficient may significantly alter CO_2_ elimination (*P* < 0.001) (Fig. 4 and *Supplemental Digital Content 5*)*.* For each supplementary unit of decarboxylation index (*expressed in L*^*2*^*/min*), CO_2_ elimination increased 5.2 times less under a *DISTAL DRAINAGE* configuration than under a *CENTRAL DRAINAGE* configuration (*P* < 0.001). Compared to the *central jugular-jugular* configuration, the CO_2_ elimination by unit of decarboxylation index was significantly lower under the other configurations: −0.3mL(STPD)/min (CI 95% −1.3‒0.7 mL(STPD)/min) with *central femoro-femoral* configuration (*P* = 0.60), −3.2 mL(STPD)/min (CI 95% −4.4‒ −2.1 mL(STPD)/min) with *distal jugular-jugular* configuration (*P* < 0.001), and −5.2 mL(STPD)/min (CI 95% −7.0‒ −3.4 mL(STPD)/min) with *distal femoro-femoral* configuration (*P* < 0.001). Based on the same phenomenon, the higher the R coefficient, the lower the CO_2_ elimination by unit of decarboxylation index was, with a significant difference for values of R coefficient of 35% or more (*P* < 0.001; *Supplemental Digital Content 5*).

### ECCO_2_R weaning, specific complications and outcome

**Fig. 5:**
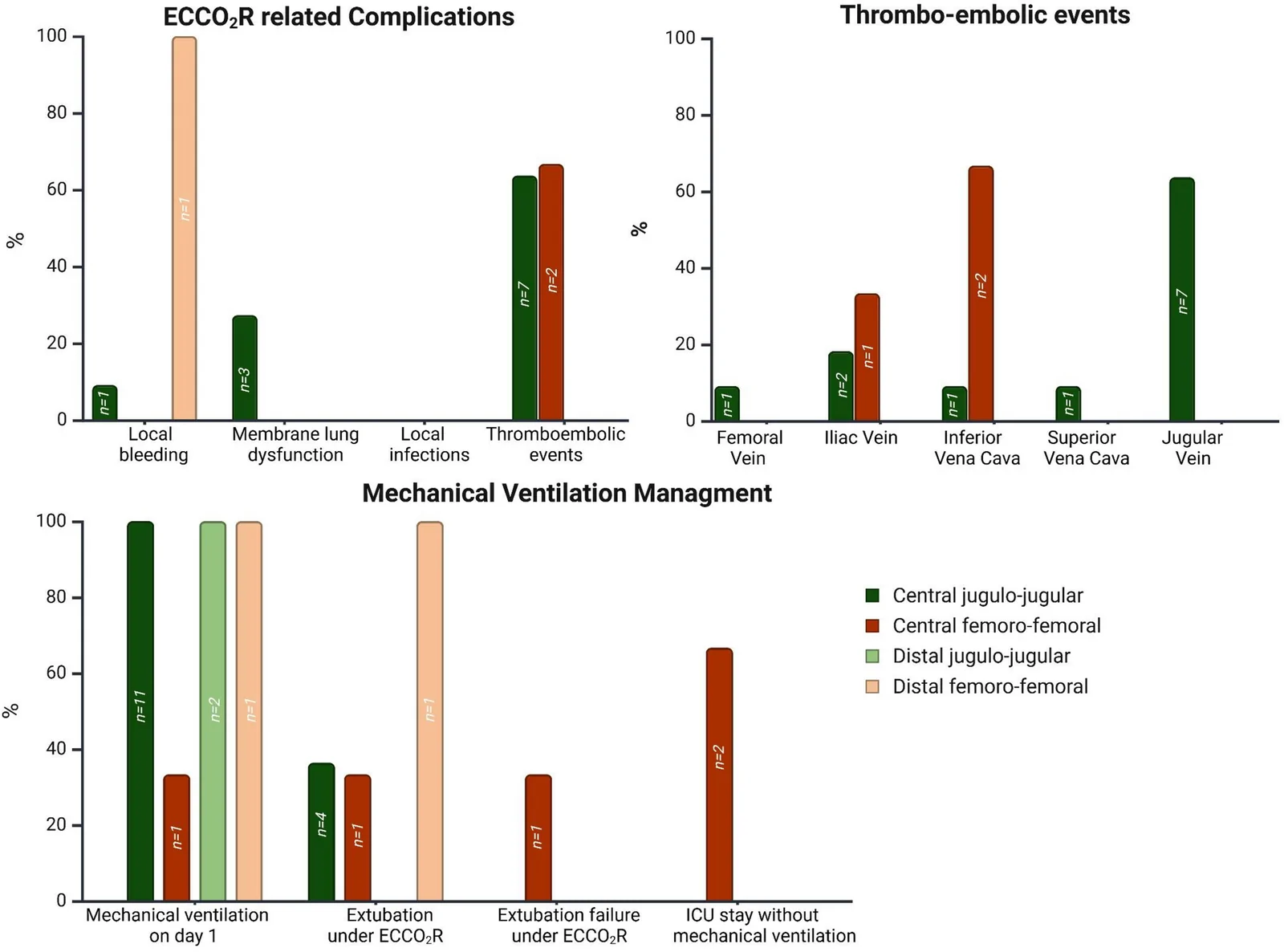
Complications and outcome. Two-thirds of CENTRAL DRAINAGE configuration were associated with cannula-associated deep vein thromboses, whether in central jugular-jugular or central femoro-femoral configurations

Following ECCO_2_R implantation, two patients required VV-ECMO support due to a delayed refractory hypoxemia (*Supplemental Digital Content* 6). Overall, 14 patients (82%) were successfully weaned from extracorporeal respiratory support, whereas 3 (17%) died while receiving high-flow ECCO_2_R therapy. The median duration of high-flow ECCO_2_R support was 8 days (IQR, 3.5–13.5 days). No major cannulation incident or local infection was observed in our cohort. Specifically, 9 patients (53%) were diagnosed with one or more thromboembolic events (Fig. 5). All occurred in patients with a central drainage configuration and consisted of cannula-associated deep vein thromboses. In contrast, neither pulmonary embolism nor septic thrombophlebitis was observed. Mechanical ventilation weaning, main complications and global outcomes are detailed in the *Supplemental Digital Content* 6.

## Discussion

We report the clinical experience of four different configurations with central or distal drainage for our cohort of 17 patients hospitalized in the ICU and treated by high-flow ECCO_2_R. All these configurations used an ECMO device, a large oxygenator surface (1.3 or 1.9 m^2^), and two single-lumen ECMO cannulas with an ipsilateral insertion, enabling all cases to reach an extracorporeal blood flow higher than 2 L/min with a consistent CO_2_ elimination. *CENTRAL DRAINAGE* configurations were demonstrated to have a low recirculation phenomenon leading to a low alteration of extracorporeal decarboxylation. In contrast, *DISTAL DRAINAGE* configurations had higher R coefficients and lower extracorporeal blood flows, leading to a significant alteration of extracorporeal decarboxylation. No CO_2_ elimination value with a distal drainage reached 110 mL(STPD)/min, explaining the potential clinical limitations of these configurations when a significant decarboxylation dose is required.

Although ECCO_2_R therapy carries important potential for the management of acute respiratory failure to avoid or reduce mechanical ventilation toxicity, its effectiveness remains difficult to demonstrate [18]. One of the main explanations is probably the insufficient decarboxylation by many devices or configurations. One major determinant of extracorporeal decarboxylation is indeed extracorporeal pump flow, requiring in many clinical situations values close to 2 L/min, while most devices and configurations cannot reach more than 1 L/min [10, 14, 18, 19]. In previous work, we indeed demonstrated a linear relationship between CO_2_ elimination and extracorporeal pump flow beyond 2 L/min [19]. Although CO_2_ elimination value does not directly translate into a clinically perceptible change at the bedside, it is a key surrogate marker reflecting the ability of the extracorporeal device to reduce PaCO_2_, minute ventilation, and/or respiratory workload [19]. However, our clinical management strategy for the study patients was not guided by this physiological parameter, which should be taken into account when interpreting our results. Regardless, our unit protocol differs from standard ECCO_2_R practice by using two ECMO single-lumen cannulas, allowing independent selection of the drainage and reinfusion sites. The two cannulas may be inserted on the same site (ipsilateral insertion), reducing the number of venous access sites required for this procedure. Our study demonstrates thus that proximal positioning of the draining cannula provides more reliable delivery of high and stable extracorporeal pump flow during extracorporeal respiratory support.

Otherwise, even if the value of extracorporeal blood flow is a key determinant guaranteeing the decarboxylation dose under ECCO_2_R support, optimizing this parameter is not enough. The present study demonstrates indeed that CO_2_ elimination was directly impacted by the type of configuration, as well as by the importance of R coefficient. The R Coefficient remains inherently dependent on the assumed SvO_2_ and should therefore be interpreted with appropriate caution. However, a significant R coefficient necessarily results in a marked reduction in effective extracorporeal blood flow. In contrast, when the phenomenon of extracorporeal recirculation is low, R coefficient < 35% in our cohort, no clinical consequences were visible. Therefore, distal drainage configurations were clearly associated with high R coefficients in our cohort, reducing their ability to remove carbonic gas. Although expected, this result makes their clinical relevance debatable. We assume indeed that the distal drainage configurations used in our cohort have similar characteristics to single femoral or jugular dual-lumen cannulas, which are used in most ECCO_2_R configurations in clinical practice [21]. Thus, several factors may explain the poor performance observed with distal drainage in our series. Poor performance that we hypothesize to be comparable to that of single dual-lumen cannulas widely used by many teams for ECCO_2_R strategies. First, the rheological conditions of extracorporeal drainage are not optimal in the distal portions of the *inferior* or *superior vena cava*. The impedance there is increased due to many factors: low available blood volume, possible occlusion of side holes by the venous wall, and reduction of suction pressure gradient induced by negative pressures related to spontaneous breathing [22]. Second, because venous blood return is lower in the distal portions of the *inferior* or *superior vena cava*, the proportion of blood drainage coming from extracorporeal reinfusion will rapidly become dominant. This logically leads to a high extracorporeal recirculation and a reduced effective extracorporeal blood flow [23]. Third, the reduction of inlet values of CO_2_ content induced by the high recirculation reduces the potential of diffusion of CO_2_ across the membrane followingFick’s law and therefore CO_2_ transfer [11, 24].

Our work shows therefore the relevance of the dissociation of drawing and reinfusion areas under ECCO_2_R therapy allowed by the use of two single-lumen cannulas. A central draining cannula provides more efficient venous drainage and captures a larger proportion of CO_2_-rich blood, despite the increased resistance associated with its greater length. Contrary to expectations, distal positioning of the reinfusion cannula has only a limited impact. The *central jugular-jugular* configuration implemented in the present study follows the *inferior vena cava*-to-*right atrium* route. This route, the most commonly used under VV-ECMO, has the best performance in terms of reduced recirculation and optimized drainage conditions. Nevertheless, our experience led us to believe that the jugulo-jugular approach has several limitations during ECCO_2_R implantation, including the need for sedation and longer placement times due to the requirement for ultrasound guidance to advance the drainage cannula across the right atrium. Placement of this kind of configuration in awake patients seems to us thereforeless advisable. In addition, the jugular site slightly forces head rotation due to the size and rigidity of the tubing, which may be uncomfortable for an awake patient, particularly during the respiratory weaning. On the other hand, the central femoro-femoral configuration has been shown to be reliable in our patients, providing high and stable extracorporeal blood flow with acceptable recirculation. Proximal placement in the *inferior vena cava* near or into the right atrium, gives optimal drainage conditions in our experience that are not influenced by the respiratory cycle. The observable recirculation phenomenon non-significantly alters the decarboxylation performance. This configuration seems to us easier to implement in awake patients using local anesthesia or simple sedation. Moreover, counterintuitively, the seated position with this femoral configuration does not impair extracorporeal flow dynamics or blood flow, owing to the use of a wire-reinforced drainage cannula and its central positioning. Respiratory consolidation and non-invasive ventilation therefore appear to us to be particularly interesting with this configuration (Table 2).

**Table 2:** Cannulas characteristics for each ECCO_2_R configuration

	Draining cannula		Reinfusion cannula	
	Brand—diameter/length	n	Brand—diameter/length	N
Central Jugulo-jugular configuration	Maquet®—19 FR/38 cm	8	Maquet®—15 FR/15 cm	7
	Maquet®—21 FR/38 cm	2	Edwards Lifescience®—16 FR/15 cm	4
	Maquet®—23 FR/38 cm	1		
Central femoro-femoral configuration	Edwards Lifescience®—20 FR/65 cm	3	Maquet®—15 FR/15 cm	3
Distal jugulo-jugula configuration	Maquet®—17 FR/15 cm	1	Maquet®—15 FR/15 cm	1
	Maquet®—17 FR/15 cm	1	Maquet®—15 FR/15 cm	1
Distal femoro-femoral configuration	Maquet®—19 FR/38 cm	1	Maquet®—15 FR/15 cm	1

The only serious adverse events related to ECCO_2_R reported in our cohort were a high rate of deep vein thrombosis, mainly at the jugular site. Two-thirds of our patients under *CENTRAL DRAINAGE* configurations were indeed diagnosed with deep vein thrombosis. These adverse events, which did not induce any consequences in our population, raise however the question of the risk–benefit ratio of this technique. It is probable that our anticoagulation strategy was insufficient. We suppose that a higher degree of hypocoagulation should be targeted. The systematic use of CT scans following ECCO_2_R weaning to identify these specific complications may moreover provide a more comprehensive description of thrombosis occurrence, whereas other series relying on clinically driven detection may underestimate the number of thromboses. Furthermore, while simplifying the ECCO_2_R configuration, the choice of ipsilateral placement may influence thrombosis occurrence by altering venous flow. Notably, deep vein thrombosis was only observed following ECCO_2_R explantation, raising questions regarding the adequacy of anticoagulation. These cannula-associated deep vein thrombosis are generated by the activation of the coagulation cascade from endothelial injury upon decannulation. A percutaneous closure system could reduce this kind of complication [25, 26]. We also assume that the hypocoagulation state level is particularly important during the decannulation phase.

In invasive ECCO_2_R therapy such as ours, one could fear the risk of respiratory worsening and the need for EMCO therapy, considering that implantation sites are already used. Contrary with the concern, ECCO_2_R therapy using two single-lumen cannulas allows a simple shift/change toward a VV ECMO therapy in case of refractory hypoxemia by a change of configuration: a V-VV configuration in the jugular-jugular ECCO_2_R configuration (see *Supplemental Digital Content* 6) and a VV-V configuration in the femoro-femoral ECCO_2_R configuration.

Our study has several limitations. First, it is a single-center retrospective design, with a limited number of patients, providing limited extrapolation and an underpowered analysis of safety. However, intra- and inter-individual variance in each group was very low. Reasonably, baseline characteristics of each group cannot be reliably established and therefore should not be compared. Second, the marked imbalance between groups limits a reliable assessment of distal drainage performance. Six measures for each patient were however analyzed, which provides a meaningful number of observations despite the limited number of patients. Third, the low number of distal configurations likely reflects prior clinical preference and therefore should be considered a selection bias. These patients also had higher admission SAPS II scores. Fourth, our analysis focused only on the first 72 h of ECCO_2_R therapy. The membrane alteration that progressively occurs throughout the days under extracorporeal respiratory support probably influences our conclusions. Fifth, SvO_2_ value was determined a priori at 65%, which may induce a classification bias. However, the actual value of SvO_2_ is known to be unavailable under extracorporeal respiratory support since extracorporeal reinfusion inevitably increases oxygen saturation in the cava system and the right atrium. An estimation was therefore necessary. Moreover, inlet PaCO_2_ in our series followed the same trend as the calculated inlet SvO2 (*Supplemental Digital Content* 3), demonstrating a similar increase with the recirculation coefficient. Sixth, all extracorporeal therapy should aim to reduce the need for mechanical ventilation, its toxicity, or its duration. Our study does not address, however, these clinical outcomes since it focused on technical and performance aspects that seemed relevant. Seventh, two oxygenators with different membrane surface areas were used; however, one device was employed less frequently. In our study, membrane surface area did not emerge as a significant determinant of performance, although its influence could not be reliably assessed in our analysis and therefore cannot be excluded. Finally, minor design updates, manufacturing variability, or subtle changes in materials and devices may have occurred over the study period, even though no major modifications were declared by the manufacturers. This could slightly influence our results and configuration performance.

## Conclusion

In conclusion, we report the feasible and effective clinical experience of four different configurations of high-flow ECCO_2_R therapy, using an ECMO device, two single-lumen cannulas, and ipsilateral cannulation. This technical choice enables the dissociation of drainage and reinfusion areas. The draining cannula may thus be precisely placed in a central or distal position. Central drainage configurations demonstrated higher extracorporeal pump flows and low extracorporeal recirculation, leading to better CO_2_ elimination performance. In contrast, distal drainage configurations, relatively similar to usual single dual-lumen cannulas, appear to be associated with a high recirculation phenomenon causing a strong alteration of decarboxylation.

## Supplementary Information

Below is the link to the electronic supplementary material.

Supplementary file1 (DOCX 1139 kb)

## Data Availability

Dr Charbit had full access to all of the data in the study and takes responsibility for the integrity of the data and the accuracy of the data analysis. The datasets used and/or analysed during the current study are available from the corresponding author on reasonable request.
