# Supplementary material for: Impact of draining cannula position and configuration under high-flow extracorporeal CO2 removal (ECCO2R) on recirculation and extracorporeal decarboxylation
Source: J Artif Organs. 2026 Oct 10;29(4):69. doi: 10.1007/s10047-026-01582-7 (PMC13653881; doi:10.1007/s10047-026-01582-7)
Supplement: Supplementary file 1 [file 10047_2026_1582_MOESM1_ESM.docx]

**SUPPLEMENTAL DIGITAL COMPONENT 1. Study equations**

The decarboxylation index (DI) was defined as the product:

Decarboxylation Index = Q_EC_ × GF *where Q_EC_ and GF are in L.min^-1^, DI is in expressed in L².min^-^²*

Recirculation (R) coefficient was determined as:

S_in_ – S_v_O_2_/S_out_-S_v_O_2_. *where Sin0_2_, SvO2, Sout and R coefficient are in %*

*Because S_v_O_2_ could not be reliably available under extracorporeal respiratory support, a value of 65% was considered as default*.

Effective extracorporeal blood flow was defined as follows:

Effective Q_EC_ = Q_EC_ × (1-R coefficient) *where Q_EC_ and effective QEC are in L.min^-1^, R ceofficient is in %*

Inlet-arterial gradient of partial pressure of carbon dioxide was defined as follows determined for each pair of samples:

Δ_(in-art)_ PCO_2_ = P_in_CO_2_ – P_art_CO_2_ *_where_* *_PCO2 is in mmHg_*

Total CO_2_ content (_ct_CO_2_) was determined from blood samples using the simplified equation, established by Douglas *et al.*^1^, extrapolates the erythrocyte CO2 component more simply:

_ct_CO_2_ = plasma _ct_CO_2_ × ( 1 − 0.0179 × Hb/[3.352 − 0.456 × SO_2_] × [8.142 − pH] )

_ct_CO_2_ =	(1 − Hb/33.8) × 0.0307 × P CO_2_ × (1 + 10^pH − pKp^) × 2.226 × (1 − 0.0179 × Hb/ [3.352 − 0.456 × SO_2_] × [8.142 − pH] )

*where ctCO2 is in mL.dL^-1^ of blood, Hb is in g.dL^-1^ and P CO2 is in mmHg. pK_p_ = 6.125 − log(1 + 10[ pH − 8.7])*

CO_2_ elimination representing the amount of extracorporeal CO_2_ eliminated was calculated using the following equation:^21,22^

CO_2_ elimination = (ctCO_2-in_ – ctCO_2-out_) × Q_EC_ × 10	*where CO_2_ elimination is in mL.min^-1^ of CO_2_, ΔTM ctCO_2_ is in mL.dL^-1^ of blood, and Q_EC_ is in L.min^-1^*

_ct_CO_2_; total carbon dioxide content in blood; _ct_CO_2-in_, total carbon dioxide content in blood from inlet cannula circulation; _ct_CO_2-out_, total carbon dioxide content in blood from outlet cannula circulation; GF, fresh gas flow; P_art_CO_2_, partial pressure of carbon dioxide in the plasma of arterial gases ; P_in_CO_2_, partial pressure of carbon dioxide in the plasma from inlet circulation; Q_EC_, extracorporeal blood flow; R coefficient, recirculation coefficient; S_in_O_2_, oxygen saturation of blood in the inlet cannula circulation; S_out_O_2_, oxygen saturation of blood in the outlet cannula circulation; S_V_O_2_, oxygen saturation of blood in the pulmonary artery.

1. Douglas AR, Jones NL, Reed JW. Calculation of whole blood CO2 content. *J Appl Physiol* 65: 473–477, 1988.

**SUPPLEMENTAL DIGITAL COMPONENT 2. Characteristics of cannulas in each configuration**

| **Cannulas characteristics for each configuration** | | | | |
| --- | --- | --- | --- | --- |
| ECCO_2_R configuration | **Draining cannulas** |  | **Return cannulas** |  |
|  | ***Brand – diameter / length*** | ***n*** | ***Brand – diameter / length*** | ***n*** |
| ***Central jugulo-jugular* configuration** | Maquet^®^ - 19FR / 38cm  Maquet^®^ - 21FR / 38cm  Maquet^®^ - 23 FR / 38cm | 8  2  1 | Maquet^®^ - 15FR / 15cm  Edwards Lifescience^®^- 16FR / 15cm | 7  4 |
| ***Central femoro-femoral* configuration** | Medtronic^®^ - 21FR / 65cm  Edwards Lifescience^®^ - 20FR / 65cm | 1  2 | Maquet^®^ - 15FR / 15cm | 3 |
| ***Distal jugulo-jugular* configuration** | Maquet^®^ - 17FR / 15cm | 2 | Maquet^®^ - 15FR / 15cm | 2 |
| ***Distal femoro-femoral* configuration** | Maquet^®^ - 19FR / 38cm | 1 | Maquet^®^ - 15FR / 15cm | 1 |

*All drainage cannulas were multi-staged interrupted in the distal segment (10 cm), except for the one used in the distal jugulo-jugular configuration.*

**SUPPLEMENTAL DIGITAL COMPONENT 3. Partial pressure in CO_2_ in plasma across the extracorporeal membrane**

*Median inlet-arterial- gradient of partial pressure of carbon dioxide (P_in_CO_2_ - P_art_CO_2_) was dependent of the configuration. This gradient should be a positive value in absence of recirculation because of the higher values of PCO_2_ in venous return. It was however negative* *in DISTAL DRAINAGE configurations and when recirculation increased beyond a threshold of R coefficient close to 30%.*

P_art_CO_2_, partial pressure of carbon dioxide in the arterial gases; P_in_CO_2_, partial pressure of carbon dioxide in the plasma from inlet circulation; P_out_CO_2_, partial pressure of carbon dioxide in the plasma from outlet.

**SUPPLEMENTAL DIGITAL COMPONENT 4A. Effect of the assumed SvO₂ value on the R coefficient**

**SUPPLEMENTAL DIGITAL COMPONENT 4B. Effect of the assumed SvO₂ value on the CO_2_ elimination**

**SUPPLEMENTAL DIGITAL COMPONENT 4C. Effect of the assumed SvO₂ value on the CO_2_ elimination**

**SUPPLEMENTAL DIGITAL COMPONENT 5. Mixed linear regression analysis**

| **MODEL 1: prediction of CO_2_ elimination** *(mL STPD_*_minute^-1^)* | | | | |
| --- | --- | --- | --- | --- |
| ***Fixed effects*** | ***Β coefficient*** | ***confidence interval 95%*** | ***Type error*** | ***P value*** |
| Intercept | 48.84 | 40.72 – 56.96 | 4.14 | <0.0001 |
| ECCO_2_R configuration |  |  |  |  |
| *CENTRAL DRAINAGE* _*_ decarboxylation index | 5.17 | 4.43 − 5.91 | 0.37 | <0.0001 |
| *DISTAL DRAINAGE* _*_ decarboxylation index | 0.99 | 0.03 − 1.95 | 0.49 | 0.042 |
|  |  |  |  |  |
| *The influence of the ECCO_2_R configurations (central/distal) and the decarboxylation index on CO_2_ elimination was specifically assessed using fixed effect β coefficients, their 95% confident intervals, and their P value for Model 1. Patient, admission SAPS II, type of oxygenator, day of ECCO_2_R implementation during hospitalization were not found significant in this model. Because reference variables (CENTRAL DRAINAGE configuration) may be considered as “optimal conditions”, an alteration of CO_2_ elimination was highlighted by a reduction of the slope and a negative multivariate β coefficient.* | | | | |
| **MODEL 2: prediction of CO_2_ elimination** *(mL STPD_*_minute^-1^)* | | | | |
| ***Fixed effects*** | ***Β coefficient*** | ***confidence interval 95%*** | ***Type error*** | ***P value*** |
| Intercept | 51.14 | 41.57 − 60 73 | 4.89 | <0.0001 |
| Decarboxylation index (*L²_*_min^-2^*) | 5.96 | 5.33 − 6.60 | 0.32 | <0.0001 |
| ECCO_2_R configuration _*_ Decarboxylation index (*L²_*_min^-2^*) |  |  |  |  |
| *Central jugulo-jugular configuration* | *Ref.* | . | . | . |
| *Central femoro-femoral configuration* | -0.27 | -1.28 − 0.74 | 0.51 | 0.60 |
| *Distal jugulo-jugular configuration* | -3.2 | -4.41 − -2.11 | 0.59 | <0.0001 |
| *Distal femoro-femoral configuration* | -5.2 | -6.98 − -3.43 | 0.91 | <0.0001 |
|  |  |  |  |  |
| *The influence of the ECCO_2_R configurations and the decarboxylation index on CO_2_ elimination was specifically assessed using fixed effect β coefficients, their 95% confident intervals, and their P value for Model 2. Patient, admission SAPS II, type of oxygenator, day of ECCO_2_R implementation during hospitalization were not found significant in this model. Because reference variables (Central jugulo-jugular configuration) may be considered as “optimal conditions”, an alteration of CO_2_ elimination was highlighted by a reduction of the slope and a negative multivariate β coefficient.* | | | | |
| **MODEL 3: prediction of CO_2_ elimination** *(mL STPD_*_minute^-1^)* | | | | |
| ***Fixed effects*** | ***Β coefficient*** | ***confidence interval 95%*** | ***Type error*** | ***P value*** |
| Intercept | 51.89 | 44.26 − 59.53 | 4.14 | <0.0001 |
| Decarboxylation index (*L²_*_min^-2^*) | 5.99 | 5.36 − 6.62 | 0.32 | <0.0001 |
| R coefficient _*_ Decarboxylation index (*L²_*_min^-2^*) |  |  |  |  |
| R coefficient <20% | *Ref.* | . | . | . |
| R coefficient 20%-35% | -0.14 | -0.58 − 0.30 | 0.23 | 0.54 |
| R coefficient 35%-50% | -2.6 | -3.86 − -1.37 | 0.64 | <0.0001 |
| R coefficient 50%-75% | -3.9 | -4.99 − -2.90 | 0.53 | <0.0001 |
| R coefficient >75% | -5.5 | -7.24 − -3.86 | 0.86 | <0.0001 |
|  |  |  |  |  |
| *The influence of the ECCO_2_R configurations and the decarboxylation index on CO_2_ elimination was specifically assessed using fixed effect β coefficients, their 95% confident intervals, and their P value for Model 3. Patient, admission SAPS II, type of oxygenator, day of ECCO_2_R implementation during hospitalization were not found significant in this model. Because reference variables (Central jugulo-jugular configuration or R coefficient <20%) may be considered as “optimal conditions”, an alteration of CO_2_ elimination was highlighted by a reduction of the slope and a negative multivariate β coefficient.* | | | | |

**SUPPLEMENTAL DIGITAL COMPONENT 6A. Complications and outcomes of study patients**

| Early respiratory impairment | Following ECCO_2_R implantation, two patients required a VV-ECMO support due to a delayed refractory hypoxemia (one with a central jugular-jugular configuration and the other with distal jugular-jugular configuration). Two switches were performed using a new drainage cannula to obtain a V-VV configuration. Median duration of high-flow ECCO_2_R in the cohort was 8 days (IQR, 3.5‒13.5 days). |
| --- | --- |
| ECCO_2_R weaning | A total of 14 patients (82%) could be weaned from extracorporeal respiratory support, while 3 (18%) died under high-flow ECCO2R therapy. Median duration of high-flow ECCO2R in the cohort was 8 days (IQR, 3.5‒13.5 days). |
| Mechanical ventilation weaning | Mechanical ventilation needs during ICU stay were as follows: two patients (12%) did not require mechanical ventilation during their hospitalization; others (88%) were ventilated before ECCO_2_R implementation. Five patients (29%) experienced a successful mechanical ventilation weaning under high-flow ECCO_2_R, while the 10 others (59%) underwent an attempted prior ECCO_2_R weaning. A tracheotomy was used in four patients (23%) for a difficult respiratory weaning. No extubation failure was observed. Median duration of mechanical ventilation was 16 days (IQR, 5‒38 days). |
| Specific complications | No major cannulation incident or local infection was observed in our cohort. Two incidents of bleeding around cannulas required external compression and interruption of heparin, with no other consequences. Extracorporeal membrane dysfunction occurred in three cases. These biological alterations spontaneously resolved with a change of extracorporeal lung membrane (on days 5, 17, and 20, respectively). Nine patients (53%) had diagnosis of one or more thrombo-embolic events (Figure 5). All of them had a central drainage configuration. Sixty-four percent (7/11) of central jugular-jugular configurations and 66% (2/3) of central femoro-femoral configurations experienced cannula-associated deep vein thromboses. No pulmonary embolism and no septic thrombophlebitis occurred in our cohort. |
| Outcome | Five patients (29%) died: two had been weaned from high-flow ECCO_2_R, whereas the three others were still under ECCO_2_R therapy. Two deaths were due to multi-organ failure, two due to septic shock, and one due to anaphylactic shock. The median durations of ICU and hospitalization in our cohort were 32 days (IQR, 12‒49 days) and 46 days (IQR, 18‒51 days), respectively. |

**SUPPLEMENTAL DIGITAL COMPONENT 6B. Switch from a ECCO_2_R to a V-VV ECMO configuration**

*Starting from jugulo-jugular configuration, the two jugular cannulas have to be removed until their tip be positioned into the superior vena cava and linked by an Y-piece connector. An additional cannula is afterward placed for the drainage into the inferior vena cava via the femoral vein. V-VV ECMO is then operational following the inferior vena cava to right atrium route.*
